# Dental Environmental Noise Evaluation and Health Risk Model Construction to Dental Professionals

**DOI:** 10.3390/ijerph14091084

**Published:** 2017-09-19

**Authors:** Kuen Wai Ma, Hai Ming Wong, Cheuk Ming Mak

**Affiliations:** 1 Department of Paediatric Dentistry, Faculty of Dentistry, The University of Hong Kong, Hong Kong, China; paulmkw@hku.hk; 2 Department of Building Services Engineering, The Hong Kong Polytechnic University, Hong Kong, China; cheuk-ming.mak@polyu.edu.hk

**Keywords:** noise in dental environment, noise exposure assessment, psychoacoustics approach, health risk assessment, health risk model

## Abstract

Occupational noise is unavoidably produced from dental equipment, building facilities, and human voices in the dental environment. The purpose of this study was to investigate the effect of occupational noise exposure on the dental professionals’ health condition. The psychoacoustics approach noise exposure assessment followed by the health risk assessment was carried on at the paediatric dentistry clinic and the dental laboratory in the Prince Philip Dental Hospital of Hong Kong. The A-weighted equivalent sound level, total loudness, and sharpness values were statistically significantly higher for the noise at the laboratory than that at the clinic. The degree of perceived influences and sharpness of noise were found to have the impacts on the dental professionals’ working performance and health. Moreover, the risk of having a bad hearing state would a have 26% and 31% higher chance for a unit increment of the short-term and long-term impact scores, respectively. The dental professionals with the service length more than 10 years and the daily working hours of more than eight showed the highest risk to their hearing state. The worse the hearing state was, the worse the health state was found for the dental professionals. Also, the risk of dissatisfaction would be increased by 4.41 and 1.22 times for those who worked at the laboratory and a unit increment of the long-term impact score. The constructed health risk mode with the scientific and statistical evidence is hence important for the future noise management of environmental improvement.

## 1. Introduction

Occupational noise is defined as any unwanted sound being produced in working environments [1]. The international standard of the eight-hour daily occupational exposure to noise is no more than 85 dB(A) A-weighted equivalent sound level (*L*_Aeq_) for a five-day workweek in any working environment [2]. If it was over this limit, the occupational noise exposure would be a potential hazard to our hearing ability in causing noise-induced hearing loss (NIHL) [3]. Even though occupational noise exposure was within the limit, a poor sound quality of noise would bring the negative impacts of working performance [4], physiological [5], and psychological conditions, and a self-reported state of health [6] on the people in the environment. The negative symptoms of sleeping problems, fatigue, headache [7], irritation, dissatisfaction on the life [8], hypertensive heart diseases [9], and tinnitus [10] were also found to be related to the noise exposure. In the dental environment, occupational noise can be generated from dental equipment, building facilities, and human activities, such as the sounds from high speed and low speed handpieces, suction tubes, ultrasonic scalers, air-conditioning systems, broadcasting systems, computers, human voices in conversation, and children crying. Previous studies showed that the sound pressure level (SPL) of the noise from the dental equipment used for dental treatments at dental clinics and the dental engines used for precast teeth model preparations at dental laboratories could be over 70 dB(A) and 90 dB(A) [11], respectively. The necessity for dental professionals to use such noisy dental equipment close to them hence increased their prevalence of hearing impairment from the long-time occupational exposure [12]. Moreover, a high energy content was found at the high frequency components of the noise [13] from the results of their SPL spectrum analyses. It might account for why the NIHL of the dental professionals at a higher frequency range was found to be heavier [14] in spite of less than 85 dB(A) [15,16] at the dental environment. However, the acoustic metrics measurement in the traditional research on noise can only reveal the acoustical properties of sounds, it is not adequate to explain the perceptual processes of listeners. The combination of acoustic metrics and psychoacoustic metrics was one of the possible ways of measuring the sound characteristics of noise in more detail [17]. In addition, the integrated understanding of the associations between the objective properties of sounds, and listeners’ perceived sound quality and meaning of noise, quality of life, and behaviour changes from the environment was necessary in providing a more audible safety environment [18]. The studies showed that the sound quality of dental noise was associated with the negative psychological responses such as dental anxiety in patients [19,20,21], and annoyance [22] and other influences [23] on dental professionals. Hence, a health risk assessment is essential to measure the health conditions of the dental professionals from their occupational exposure to a bad sound quality noise. Statistical analyses could then integrate the objective data from the acoustic and psychoacoustic metrics with the subjective data from the measurements on the perceived noise influences, negative impacts, and health conditions of the dental professionals in understanding their associations. It is important for the prevention of negative impacts on the dental professionals from providing a better hospital strategy planning and appropriate noise control works.

According to the equal loudness contour plot in the International Organization for Standardization (ISO) standard [24] of equal-loudness contours, the density of the curves above the threshold of audibility at low frequencies is higher than that at high frequencies. It means the loudness response of low frequency sounds for a same increment of SPL above the threshold of audibility is more vigorous when compared to that of high frequency sounds. In the A-weighted SPL calculation, the SPL independent low weighting will be applied to the low frequency components of sounds. The A-weighted SPL and *L*_Aeq_ of low frequency noise then, hence would be underestimated in their calculation. Also, the sound characteristics cannot be fully explained by the *L*_Aeq_ value of noise. Thus, the sound quality analysis with psychoacoustics approach was appended to the noise exposure assessment in the study. The psychoacoustics metrics calculation of “total loudness (*N*)”, “specific loudness (*N*’) spectrum”, and “sharpness” were supplemented with *L*_Aeq_ and SPL in 1/3-octave band spectrum to measure the sound characteristics of the dental noise. Although both the values of *L*_Aeq_ and *N* approximate the human loudness responses to sounds, the additional consideration of the transmission characteristics of middle ear is included in a loudness calculation [25]. The loudness sensation from the excitation on basilar membranes of ears is estimated by the calculation of the excitation above the threshold of audibility at different critical bands in Bark scale. The audible frequency range of human can be divided into total 24 critical bands in the Bark scale. The loudness sensation caused by the sound components in different critical bands is showed in the corresponding values of *N’* in the unit of sone/Bark. The total loudness sensation caused by all the sound components in the audible frequency range hence be computed by adding up all *N’* in 24 critical bands. It means that the contribution of the sound components in either low or high frequency is included in the calculation of *N*. In contrast to an outdoor environment, low frequency noises, such as human voices, cannot be ignored in an indoor environment. The necessity of the communication between dental professionals and that with patients would be a source of the occupational noise in low frequency and cause influences on others. Especially in paediatric dentistry, the occupational noise problem from human voices is worse due to the additional noise from children cry, shout, and yell. The application of *N’* spectrum analysis hence provided the better understanding of how the loudness of the dental noise can be produced from the different noise sources in the dental environment. In the same time, sharpness metric is a quantity representing the amount of the negative feeling to high frequency noise by applying a filter to the *N’* spectrum of the noise. The comparison of the sound quality of the high frequency dental equipment noise then is helped by the quantification of the high frequency content of the noise.

The objectives of the study were to conduct the noise exposure assessment on dental professionals’ daily working environment and to have the corresponding health risk assessment of the dental professionals’ health condition. The assessments in the study were carried out at the Prince Philip Dental Hospital (PPDH), which is the only dental hospital in Hong Kong. The sound quality of the noise at the paediatric dentistry clinic and the dental laboratory of PPDH was assessed by the inducing *L*_Aeq_, SPL spectrum, *N*, *N’* spectrum, and sharpness analyses. While the influences from the dental noise, the negative impacts about working performance, short-term, and long-term impacts on the dental professionals, and the dental professional’s demography information, state of satisfaction, hearing, and health, were recorded by the self-administrated questionnaire in assessing their health condition. Besides, the different statistical analyses were applied to test the associations between the sound quality of the noise and the dental professionals’ health condition. It was of importance for the dental hospital to design their environmental management to their staff.

## 2. Materials and Methods

### 2.1. Noise Exposure Assessment

A total of 60 noise exposure assessments were carried out at the paediatric dentistry clinic and the dental laboratory in PPDH of Hong Kong. Half of the assessments were at the clinic and the others were at the laboratory. One normal working day was randomly chosen to perform the noise exposure assessments. All of the assessments were carried out within the daily working hours of dental professionals in the locations. Since dental professionals would move from place to place during their daily work, the general acoustic environment of the clinic and laboratory in the study was represented by averaging the objective data in the noise exposure assessments at the different locations instead of a single assessment at a certain location with a long elapsed time. In order to avoid the situations of too few locations or too short recording interval be measured at each location, the assessments with about 10-min elapsed time was conducted at the locations. In addition, the assessments were taken at about 1 m above the ground as the height of the ears of seated dental professionals. An advanced, 2-channel, handheld analyzer (Type 2270; Bruel & Kjaer, Naerum, Denmark) with the sound level meter software designed for environmental noise assessment and monitoring, and occupational noise evaluation was used in the study. The internal frequency analysis software would also provide a real-time analysis of the Z-weighted (“Zero” frequency weighting) or A-weighted (frequency weighting of a human ear response) SPL of noise in 1/3-octave band spectrum that covered all the audible frequencies. The averaged SPL of the noise over the elapsed time of the measurement could also be computed in the form of Z-weighted equivalent sound levels (*L*_Zeq_) or *L*_Aeq_. For the psychoacoustics metric calculation (see Appendix A), *N’* spectrum calculation in 24-Bark critical bands was based on the Loudness Model as stated in the standard ISO532B [26]. The conversion of *L*_Zeq_ in 1/3-octave band spectrum into *N’* in 24-Bark band spectrum was performed by using MATLAB (MATLAB R2017a, The MathWorks, Natick, MA, USA, 2017). The total loudness and sharpness [27] of the noise were then calculated from the calculated *N’* in the 24 critical bands. A loudness of a reference sound in 1000 Hz with 40 dB SPL is 40 phons or 1 sone. A sone value that is double in number means that the perceived loudness is twice as much. The sharpness of a sound in 1000 Hz with 60 dB SPL is 1 acum.

### 2.2. Health Risk Assessment

An ethics approval of the study was obtained from the Institutional Review Board of the University of Hong Kong/Hospital Authority Hong Kong West Cluster (UW 14-010). An informed consent from the dental professionals was obtained prior to any measurement in the study. A self-administrated questionnaire survey was included in the health risk assessment of the study. After every noise exposure, the questionnaires were personally distributed to and collected from the dental professionals at the locations. The dental professionals with any known hearing problem in the verbal questioning to them would be excluded in the study. The aim of the questionnaire in the study was to provide the assessment about the perceptual responses of dental professionals as well as their health conditions. The previous studies showed the effects of noise on subjects’ sensation to different noise sources [4], health-related symptoms [8], and self-reported health state [28] could be assessed from the subjects’ ratings in the questionnaires. Therefore, the 31-question questionnaire in the study was composed of four parts about the dental professionals’ demographic information, degree of dental noise influences, degree of the negative impacts, and overall health condition (see Table 1). The five-point Likert scale questions in parts II and III were scored from 1 to 5 in representing “Not at all”, “Occasionally”, “Medium”, “Often”, and “Very often”, respectively. For the seven questions about the dental noise influences in part II, two of them were about the dental equipment noise influences, while the others were about the background noise influences on the dental professionals. The outdoor noise influences, such as traffic noise and construction noise were not included because only two narrow roads with low traffic flow and no construction site were outside PPDH. In part III, the impact of working performance, short-term, and long-term impacts on the dental professionals from the dental noise was assessed by their ratings of the 16 questions about the negative symptoms in affecting them. In part IV, the overall assessment of the included three three-point Likert scale questions about their self-rated state (good, medium, and bad) of satisfaction, hearing, and health. These three states would be the final outcomes of the health risk assessment of the study.

### 2.3. Health Risk Model from Statistical Analyses

The objective data from the acoustic and psychoacoustic metrics was not only used for the analysis of the sound quality of the general acoustic environment, but also objectively represented the recent dental noise exposure of the dental professionals. Meanwhile, the subjective data such as the dental noise influences was to measure the general perception of the dental professionals to dental noise in their working environment. The combination of the objective and subjective data then would provide a more comprehensive understanding of the noise exposure of the dental professionals. The inclusion of more than one locations in the study provided the variation of the sound quality from the different noise sources and the associated influences on the dental professional. The statistical analyses in the study were performed to find out the associations between the sound quality of the noise at the dental environment and the results of the questionnaire survey, especially the dental professionals’ health condition. All of the data in the statistical analyses was coded and analysed by the commercial package SPSS, version 23.0 (IBM Corp., Armonk, NY, USA). Independent two-sample t-tests would be performed to analyse whether there was a difference between the sound quality of the noise at the two working locations. If the objective data at the locations were not normally distributed, the Mann–Whitney *U* test (a non-parametric test) would be applied. For the questionnaire results in the part II and part III, the summated scores of the corresponded items represented the five latent variables “equipment noise influence”, “background noise influence”, “impact of working performance”, “short-term impact”, and “long-term impact”. The summated scores as the continuous variables will be used in the further statistical analyses instead of the individual item scores. The reliability of the approach would be checked by the Cronbach’s alpha reliability tests [29]. After that, the preliminary testing of the correlations between all of the variables would be analysed by bivariate correlation tests (Spearman Rank-Order Correlation). The multiple linear regressions of the negative impacts on the dental professionals in the stepwise method were then applied to find how the variables were affected from the environmental changes. Finally, the risk of the dental professionals having a bad state of satisfaction, hearing, and health was tested by the method of multiple ordinal logistic regression.

## 3. Results

### 3.1. Results of Noise Exposure Assessment

#### 3.1.1. A-Weight SPL Measurement

The *L*_Aeq_ value of noise was statistically significantly higher for the noise at the dental laboratory (*Median* (*Mdn*) = 66.2 dB(A)) than that at the dental clinic (*Mdn* = 62.2 dB(A)), *U* = 236, *p* = 0.002, *r* = 0.41. The occupational noise exposure of the dental professionals to noise at both the dental clinic and the dental laboratory was within the international standard of not more than 85 dB(A). The highest 10-min *L*_Aeq_ at the clinic and the laboratory were 74.98 dB(A) and 91.59 dB(A), respectively.

**Figure 1:**
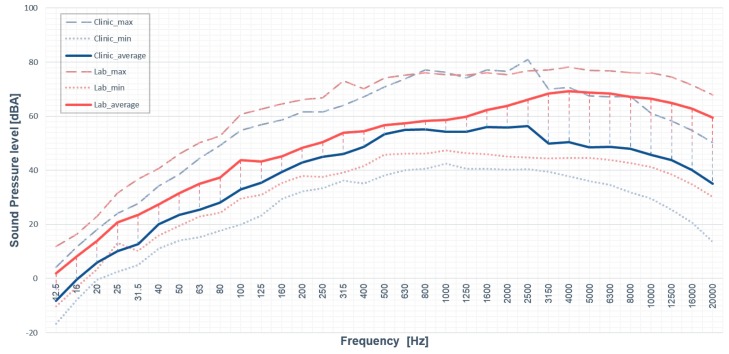
A plot of the maximum, minimum, and average A-weighted sound pressure level of the noise at the dental clinic and the dental laboratory in 1/3-octave band spectrum.

Other than the *L*_Aeq_ measurement, 1/3 octave band spectrum analysis of the noise provided a better understanding of the energy content of the noise at the two locations (see Figure 1). A similar pattern was found for the minimum A-weighted SPL spectrums of the noise at the two locations. However, the patterns of the maximum A-weighted SPL spectrums were found to be different due to the discrepancy of the dental equipment being used in the locations. The peak of the maximum A-weighted SPL spectrum of the noise at the clinic was in the frequency range from 1 kHz to 3 kHz, while that at the laboratory was in the frequency range from1 kHz to 20 kHz. Hence, a great difference between the average A-weighted SPL spectrums at the two locations in the frequency range higher than 3 kHz.

#### 3.1.2. Psychoacoustics Metrics Calculation

**Figure 2:**
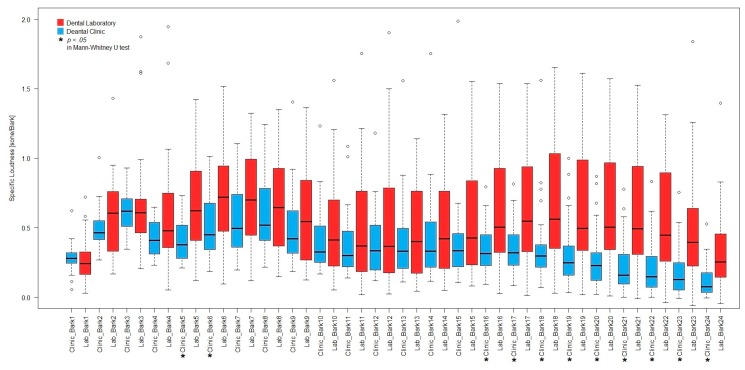
A combining boxplot of the averaged specific loudness changes of the noise at the dental clinic and the dental laboratory in 24-Bark band spectrum.

A significantly higher *N* was found for the noise at the dental laboratory (*Mdn* = 21.9 sone) as compared to that at the clinic (*Mdn* = 17.0 sone), *U* = 206, *p* < 0.001, *r* = 0.47. In addition, the averaged *N’* change of the noise at the two locations estimated the dental professionals’ loudness sensation to the generated noise during the measurement. The medians of the averaged *N’* changes in the 5th to 6th critical bands, and 16th to 20th critical bands were found to be significant higher (*p* < 0.05) at the laboratory than at the clinic (see Figure 2). The corresponding frequency ranges of those critical bands were 400 Hz to 630 Hz and 2.7 kHz to 20 kHz. Moreover, significantly higher sharpness was found for the noise at the laboratory (*Mdn* = 1.80 acum) compared to that at the clinic (*Mdn* = 1.55 acum), *U* = 231, *p* = 0.001, *r* = 0.42.

### 3.2. Health Risk Model Construction

#### 3.2.1. Demography Information

A total of 60 dental professionals completed the questionnaire survey after the noise exposure assessment near to them at the dental clinic and laboratory (see Table 2). Half of them worked at the dental clinic and the others worked at the dental laboratory. 68.3% of them were female and 31.7% of them were male. About 70% of them were over 40 years old. 70.0% of them had been worked for more than 10 years. In addition, most of them had the daily working hours of more than 8.

#### 3.2.2. Reliability of the Latent Variables

**Figure 3:**
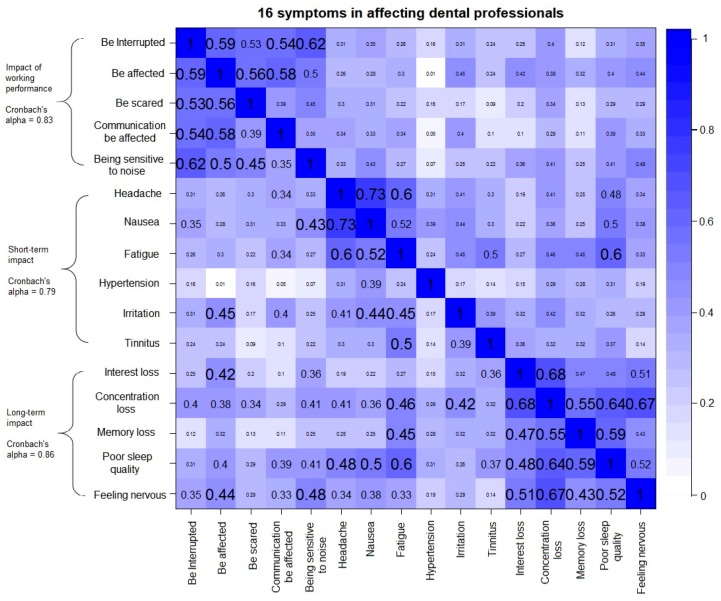
A correlation plot of the 16 symptoms in the negative impact of working performance, short-term impact and long-term impact on the dental professionals from their exposure to the dental noise.

The Cronbach’s alpha reliability coefficient indicated the internal consistency of the items. Cronbach’s alpha lager than 0.8, 0.7, and 0.6, corresponded to a good, acceptable, and questionable internal consistency of the items. The values of Cronbach’s alpha of the variables “equipment noise influence”, “background noise influence”, “impact of working performance”, “short-term impact”, and “long-term impact” were 0.86, 0.66, 0.83, 0.79, and 0.86, respectively. The high values of Cronbach’s alpha explained the reliability of the questionnaire with the consistent measurement of the concepts except the variable “background noise influence”. It also provided the evidence of using the summated scores of the items for the latent variables in the further statistical analyses. For the variable “background noise influence”, the inconsistency between the items “human voices” and “phones”, and the items “air-conditioning system”, “computers”, and “broadcasting” about the machine noise reduced the value of Cronbach’s alpha. For simplifying the further statistical analyses, all of the five items were still kept in one variable instead of being divided into different variables like background noise from human and background noise from machines. The correlations between the 16 symptoms in affecting the dental professionals were shown in Figure 3. The bluer off-diagonal box illustrated the stronger positive correlation between the corresponding variables of the row and the column. The correlation coefficients were also labeled in the plot.

#### 3.2.3. Bivariate Correlation Tests

**Figure 4:**
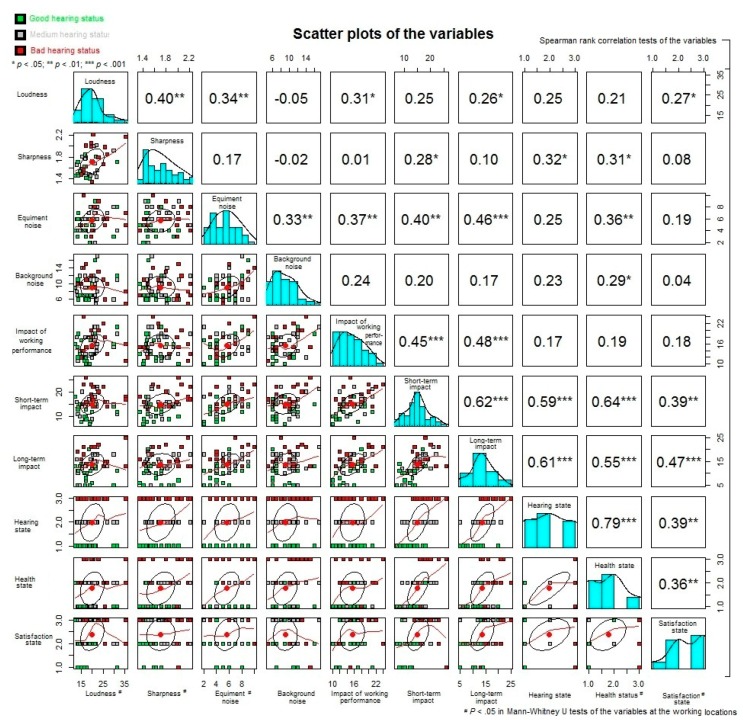
A summarized plot of the bivariate correlation test results of the variables. * *p* < 0.05, ** *p* < 0.01, *** *p* < 0.001.

The correlations between the variables in the study were first analysed by the bivariate correlation tests. All of the results of the tests were presented in the summarised plot (see Figure 4). The diagonal components of the summarised plot were the distributions of the variables. The lower panel and the upper panel of the summarised plot were the scatter plots and the correlation coefficients of the corresponding row and column variables. If the significant results were obtained in the tests, the superscript “***”, “**”, or “*” would be added next to the correlation coefficients referring to the cases of *p* < 0.001, *p* < 0.01, or *p* < 0.05, respectively. The green, grey, and red color points in the plots represented the dental professionals who had a good, medium, or bad state of hearing. The correlation ellipses and best fitting locally weighted scatterplot smoothing were also drawn in the plots. The differences between the variables loudness and sharpness at the working locations were explained in the noise exposure assessment results. Also, a number of the significant correlations were found between the sound quality of noise and the dental professionals’ health condition (*p* < 0.05). For example, sharpness of the noise was found to be correlated with the negative short-term impact on the dental professionals, and their state of hearing and health. The environmental changes such as the changes of working location, loudness of noise, sharpness of noise, and the influences from the difference noise sources, were associated with the degree of impacts on the dental professionals. Moreover, the impacts on the dental professionals would affect the dental professionals’ health condition in their self-reported sate of satisfaction, hearing, and health. The further multiple regressions between the variables hence were needed to clarify the associations between the variables.

#### 3.2.4. The Effects of the Environmental Changes to the Negative Impacts

In this section, the impact of working performance, short-term impact, and long-term impact on the dental professionals were the outcome variables in the multiple linear regressions (see Table 3), Working location, loudness, sharpness, equipment noise influence, and background noise influence were the five tested independent variables about the environmental changes in the regressions. For the regressions with a stepwise method, only the variables with a *p* less than 0.05 would remain in the models showing how the impacts were affected by the environmental changes. With a unit increment of the total score of noise influence, the score of the impact of working performance would be increased by 0.34 unit (*p* = 0.002) and the score of the short-term impact would be increased by 0.43 unit (*p* < 0.001). Furthermore, the score of the short-term impact would be increased by 0.49 (*p* = 0.013) if the sharpness of the noise increased by 0.1 acum. However, the effect of the background noise influence become less important for the long-term impact on the dental professionals. Only the dental equipment noise influence showed the significant effect on the long-term impact. The score of the long-term impact would be increased by 1.15 unit (*p* < 0.001) with a unit increment of the score of equipment noise influence.

#### 3.2.5. The Effect of the Negative Impacts to the States of Satisfaction, Hearing, and Health

Since the outcomes of the state of satisfaction, hearing, and health were ordinal. Ordinal logistic regression was hence performed to calculate the odd ratio of in a bad state against not in a bad state (see Table 4). Among all the investigated variables, the two variables “long-term impact score” and “working location”, with *p* < 0.05 remained in the final model of the dental professionals’ satisfaction state. With a unit increment of the long-term impact score, a 22% (95% CI [7%, 39%]) higher chance was found in having a bad satisfaction state. Also, the chance of having a bad satisfaction state was 0.23 (95% CI [0.075, 0.69]) times as likely for the dental professionals in the dental clinic when compared to those in the dental laboratory where the place had the poorer sound quality.

For the final model of the dental professionals’ hearing state, the four variables “short-term impact score”, “long-term impact score”, “service length”, and “daily working hours” with *p* < 0.05 remained. With a unit increment of the short-term impact score and the long-term impact score, 26% (95% CI = [4%, 53%]) and 31% (95% CI [8%, 58%]) higher chances were found in having a bad hearing state, respectively. In addition, an interaction effect was found for the service length and the daily working hours of the dental professionals on their hearing state. When compared to the dental professionals with the service length more of than 10 years and the daily working hours more than eight, the chance of having a bad hearing state was only 0.061 (95% CI [0.009, 0.41]) times as likely for those with the daily working hours of more than eight, but the service length of less than 10 years. The effect of the service length was not observed for the dental professionals with the daily working hours of less than eight. The chance of having a bad hearing state was about 0.1 times as likely for those in the daily working hours of less than eight as compared to the reference group. For the final model of the dental professionals’ health state, the two variables “short-term impact score” and “hearing state with *p* < 0.05 remained. 34% (95% CI [7%, 69%]) higher chance was found in having a bad health state with a unit increment of the short-term impact score. The chance of having a bad health state become 0.001 (95% CI [<0.001, 0.031]) and 0.068 (95% CI [0.007, 0.65]) times as likely for the dental professionals in a good or medium hear state as compared to those in a bad hearing state.

## 4. Discussion

In the results of the noise exposure assessment, the sound quality of the noise at the dental laboratory was worse than that at the dental clinic in terms of the measured *L*_Aeq_, total loudness, and sharpness of the noise. The background noise level of the noise from the building facilities without any dental equipment operation and the human voice at the two locations was indicated by their minimum A-weighted SPL spectrums of the noise. The similar pattern of the minimum SPL spectrums showed that the sound characteristics of the building facility noise at the two locations was almost the same. The floor area of the laboratory was about five times less than of the dental clinic. The difference of the spatial settings at the two locations might explain why the SPL of the noise was generally higher at the dental laboratory than that at the dental clinic. It was consistent with the finding that spatial setting would have the influence on the noise at the working environment in the previous study [30]. In contrast to the minimum SPL spectrums, the maximum SPL spectrums demonstrated the sound characteristics of the noise generated by the dental equipment operations at the locations. The high frequency noise ranged from 1 kHz to 3 kHz was found to be produced by the dental equipment operations during dental treatments from the maximum SPL spectrum at the dental clinic. It was also consistent with the results of the octave band spectrum analysis of the noise from the different dental equipment in the previous study [13]. The previous study showed the operation of ultrasonic scalers and saliva suctions, and the operation of air-rotor handpieces and triple syringes could produce the noise with high SPL in 1 kHz and at 4 kHz (covered the 31.5 kHz band in 1/3-octave band spectrum), respectively. In addition, the operation of grinders and micro motors could produce the noise with high SPL above 1 kHz. It matched with the result of the high frequency noise above 1 kHz and was found to be produced by the dental equipment operations during tooth model preparations from the maximum SPL spectrum at the dental laboratory. The discrepancy in the sound characteristics of the noise was found to come from the difference of noise sources at the two locations. Hence, the SPL difference was found for the high frequency components (>3 kHz) of the noise at the two locations from their maximum and average SPL spectrums. It also gave the evidence that the sound characteristics of the noise in the dental environment was contributed by the dental equipment operations. It also explained why the sharpness of noise was found to be significantly higher in the dental laboratory than at the clinic. Apart from the energy content distribution, the perceived loudness, as well as the region and magnitude of the excitation of the different frequency components of the noise, were estimated by the *N’* calculation in 24-Bark band spectrum. The loudness changes of the low frequency noise were hidden in the A-weighted SPL measurement due to the low weighting to the low frequency noise. The *N’* calculation of the noise at the dental environment hence provided the alternative of analysing the loudness changes contributed from different frequency components. Significantly higher *N’* changes of the noise above 3 kHz contributed from the dental equipment at the dental laboratory was consistent with the A-weighted SPL measurement results. The additional finding of the significantly higher *N’* changes of the noise ranged from 400 Hz to 630 Hz at the laboratory compared to the dental clinic were hence discovered. It means not only the dental equipment noise but also the low frequency noise like human voices could increase the loudness of the occupational noise of the dental professionals. Nevertheless, the *N* of the noise at the dental environment was mainly contributed by the *N’* of the high frequency components. Although the values of *L*_Aeq_ of the noise at the two locations were within the occupational limit to hearing loss, more attention should be paid to the high energy content of the high frequency components of the noise from the dental equipment operations, as the study showed the hearing impairment of the dental professional’s hearing ability to 3 kHz and 4 kHz sounds [31]. In addition, the higher degree of the equipment noise influence and the worse satisfaction state were observed for the dental professionals who worked at the location with poorer sound quality.

**Figure 5:**
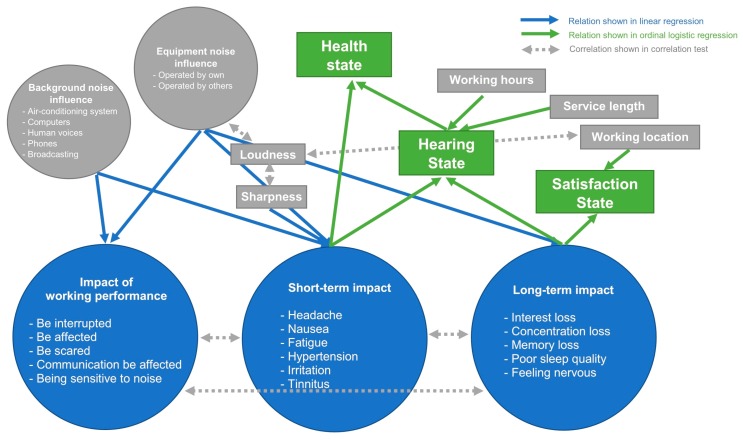
The health risk model connected the environmental changes to the dental professionals’ health condition.

The health risk assessment of the study focused on measuring the health condition of the dental professionals from their state of satisfaction, hearing, and health. The application of the statistical analyses then provided the better understanding of how the health condition of the dental professionals was affected from the environmental changes and their impacts. The final health risk model (see Figure 5) was established from the results of the statistical analyses. The double-headed arrows in the figure represented the results of the significant correlations between the variables. If a causal relationship between variables is proved by a regression analysis, a direction of the arrow in the figure will be changed to single-headed. The variables from the direct measurement were drawn in the shape of rectangle, while the latent variables from the measurements of a list of variables were shown in the shape of circle. The results of the Cronbach’s alpha reliability tests revealed the reliability of the approach in the questionnaire to assess the degree of the noise influences and the negative impacts on the dental professionals. Firstly, the correlations between the variables loudness, sharpness, equipment noise influence, and working location were well explained in the results of the noise assessment. Secondly, the noise influences from both dental equipment, building facilities, and human voices would have the direct negative impact of working performance on the dental professionals. The greater impact of working performance on the dental professionals would then be shown up in the prevalence of the symptoms of the work be interrupted and be affected, be scared, the communication be affected, and being sensitive to noise. Thirdly, the short-term impact on the dental professionals was affected by not only the dental noise, influences, but also the sharpness of noise. The prevalence of the symptoms of headache, nausea, fatigue, hypertension, irritation, and tinnitus was dependent on the energy content of the high frequency components of noise but not the overall energy content. It explained why the overall SPL change was not enough to account for the variation of the self-report parameters [28]. Fourthly, the noise influence from dental equipment was more dominant than that from building facilities and human voices in affecting the long-term impact on the dental professionals. The prevalence of the symptoms of interest loss, concentration loss, memory loss, poor sleep quality, and feeling nervous was then occurred to the dental professionals from their long-term exposure to the dental equipment noise. The dental professional’s dissatisfaction of the working environment would be eventually resulted from their long-term impact due to the dental equipment noise at their working location. Fifthly, both the negative short-term impact and long-term impacts on the dental professionals from their occupational noise exposure would increase their risk of having a bad hearing state. The risk would be further increased for those with the daily working hours of more than eight. The further increment of the risk would be found for those worked for more than 10 years with the daily working hours more than eight. Finally, the risk of having a bad health state was highly associated with the risk of having a bad hearing state. Besides, the risk of having a bad health state would be increased from the dental professionals’ prevalence of the negative symptoms of headache, nausea, fatigue, hypertension, irritation, and tinnitus from their exposure to the high frequency noise. The study results showed the consideration of both objective and subjective data in the statistical analyses provided a more comprehensive understanding of the effects of noise on the dental professionals from sound properties of noise, to their perceptual influences, and finally to their health conditions. The objective data compensated the weakness of subjective data in assessing the acoustical properties of noise, while the subjective data compensated the weakness of objective data in assessing the perceptual responses to noise.

## 5. Insights and Limitations

The monitoring for sharpness and loudness of noise is required as it can be served as the indicator in assessing the noise impacts on the dental professional in their working environment. Different from the other studies of the dental noise in using *L*_Aeq_ and A-weighted SPL spectrum analyses, this study included the psychoacoustics approach of using *N*, *N’* spectrum and sharpness analyses of the noise. Although the similar results were found between *L*_Aeq_ and *N* measurements of the overall perceived loudness of the noise, the *N’* spectrum analysis showed its ability to assess the corresponding loudness changes from the different frequency components of the noise. The capacity of identifying the perceived loudness changes from different noise sources, such as human voices, was important for the environmental noise evaluation, especially for the indoor environment. Since there was not any parameter in quantifying the noise from human voices, it limited further statistical analyses of the association between the noise from human voices and the impacts on the dental professionals. Also, more attention should be paid to the high SPL of the high frequency components as well as the high sharpness of the dental equipment noise. It is because the high sharpness of the noise was associated with the negative short-term psychological symptoms in affecting the dental professionals’ state of hearing and health. The sharpness calculation provided the more specific information of the sound quality of the noise other than the information of overall loudness. The quantification of the sound quality of the noise then helps in understanding the effects of the noise on the risk to the dental professionals’ health condition. Moreover, a further testing of the dental professional hearing ability to the different frequency sounds can be carried out to construct the additional validity of the study.

The sustained assessments of both the sound quality of noise and the prevalence of the negative symptoms of the dental professionals is required for the dental hospital environmental management according to the assessment results. For example, dental hospitals need to set the regulations for the daily noise exposure of their staff, to have the warning of using noise protective measures to the dental professionals before the use of the high sharpness and loudness dental equipment, and to provide a better working environment from adopting less noisy equipment, providing a larger workplace, and a partition of noise from others. The impact on the working performance on the dental professionals cannot be ignored, as it will have the directed influence on the quality of the treatment to patients. Since dental professionals usually have to suffer a long-time daily noise exposure in more than ten years, the strategy planning in providing a better occupational environment to them is important to reduce the noise influences and the negative impacts on them from the long-term noise exposure.

After having the general acoustical understanding of the dental environment, the more specific analysis of the respective acoustical properties of the dental equipment, indoor and outdoor noise sources, and the effects of the spatial setting to noise should be carried on in the future works. The sound sample recording appending to the noise level assessment would be a good way to provide a detailed analysis of the acoustical properties of noise, and facilitate the computation of different psychoacoustics metrics and the comparison of their accuracy. Also, the analysis of the associations between the perceptual responses such as emotional changes from the objective properties of noise would also a direction in the future works. Assessment, prediction, and control [32] are the three essential steps for the occupational environment improvement. The prediction [33] of how the sound quality of noise such as loudness and sharpness is varied with the indoor environment [34], outdoor environment [35], noise sources [36,37], and the equipment designs is essential for the noise control work development. The application of the appropriate noise control works and the policy hence can reduce the probable risks to the dental professionals’ health.

## 6. Conclusions

The results of the study showed the capacity of the psychoacoustics parameters in the quantification of sound quality of the noise and the estimation of its negative impacts on the dental professionals’ health condition. Although the values of *L*_Aeq_ of the noise at the two working locations were under the internal standard of noise exposure for hearing loss, the health risk model construction showed the high *N* and sharpness level could be the risk factors to the dental professionals’ health condition. The dental professionals’ long-term exposure to the noise in their working environment will affect their working performance and satisfaction to the work. Meanwhile, the short-term physiological symptoms of headache, nausea, fatigue, hypertension, irritation, and tinnitus were found to be associated with the sharpness of the occupational noise experienced by the dental professionals. The effects from those symptoms would eventually show in the bad hearing and health states of the dental professionals. It implied that sustained monitoring for the sound quality of noise, and the dental professionals’ heath condition hence is essential for the environmental management planning in providing a better working environment and reducing the risks of health to the dental professionals.

## Figures and Tables

**Table 1:** Summary of the questions in the self-administrated questionnaire survey.

Parts	Questions (Latent Variable)	Number of Questions	Scales
Part I: Demographic information	Gender; Working location; Age range; Service length; Daily Working hours	5	Nominal and Ordinal
Part II: Degree of dental noise influences	Operated by own; Operated by others (Equipment noise influence)	2	Five-point Likert scale
	Air-conditioning system; Computers; Human voices; Phones; Broadcasting (Background noise influence)	5	Five-point Likert scale
Part III: Degree of the negative impacts	Work be interrupted; Work be affected; Be scared by noise, Communication be affected, Being sensitive to noise (Impact of working performance)	5	Five-point Likert scale
	Headache; Nausea; Fatigue; Hypertension; Irritation; Tinnitus (Short-term impact)	6	Five-point Likert scale
	Interest loss; Concentration loss; Memory loss; Poor sleep quality; Feeling nervous (Long-term impact)	5	Five-point Likert scale
Part IV: Health condition	Satisfaction state; hearing state; health state	3	Three-point Likert scale

**Table 2:** Summary of the dental professionals’ characteristics.

Subject Characteristics	Number (*n* = 60)	Percentage
Gender		
Male	19	31.7%
Female	41	68.3%
Working location		
Dental Clinic	30	50.0%
Dental laboratory	30	50.0%
Age range		
20–30 years old	12	20.0%
30–40 years old	6	10.0%
40–50 years old	20	33.3%
50–60 years old	22	36.7%
Service length		
<10 years	18	30.0%
≥10 years	42	70.0%
Daily working hours		
<8 h	17	28.3%
≥8 h	43	71.7%

**Table 3:** Multiple linear regressions of the scores of the negative impacts on the dental professionals from the environmental changes.

Outcome Variables	Remained Variables	Estimate	Standard Error	*p*
Impact of working performance ^1^	Total score of noise influences	0.34	0.10	0.002
Short-term impact ^2^	Total score of noise influences	0.43	0.12	<0.001
	Sharpness	4.92	1.91	0.013
Long-term impact ^3^	Score of equipment noise influence	1.15	0.27	<0.001

^1^ *R*^2^ = 0.16, *F*(1, 58) = 11.0, *p* < 0.01; ^2^ *R*^2^ = 0.52, *F*(2, 57) = 10.7, *p* < 0.001; ^3^ *R*^2^ = 0.49, *F*(1, 58) = 18.1, *p* < 0.001.

**Table 4:** Ordinal logistics regressions of the dental professionals’ satisfaction, hearing, health state the other variables.

Outcome Variables	Remained Variables	β ^#^ (SE)	Odds Ratio (95% CI)	*p*
Satisfaction state	Long-term impact score	0.20 (0.067)	1.22 (1.07–1.39)	0.004
	Working location			
	Dental clinic	−1.48 (0.57)	0.23 (0.075–0.69)	0.009
	Dental laboratory ^^^			
Hearing state	Short-term impact score	0.23 (0.099)	1.26 (1.04–1.53)	0.006
	Long-term impact score	0.27 (0.096)	1.31 (1.08–1.58)	0.021
	Service length (Daily working hour)			
	<10 years (<8 h)	−2.34 (0.94)	0.10 (0.015–0.61)	0.013
	<10 years (≥8 h)	−2.80 (0.97)	0.061 (0.009–0.41)	0.010
	≥10 years (<8 h)	−2.47 (0.96)	0.084 (0.013–0.55)	0.004
	≥10 years (≥8 h) ^^^			
Health State	Short-term impact score	0.30 (0.12)	1.34 (1.07–1.69)	0.010
	Hearing state			
	Good	−6.59 (1.59)	0.001 (<0.001–0.031)	<0.001
	Medium	−2.68 (1.15)	0.068 (0.007–0.65)	0.019
	Bad ^^^			

Note: ^^^ Reference groups. ^#^ Testing of ln(P(bad)/(1-P(bad))) = β_0_ + β_1_X_1_ + β_2_X_2_ + … + β_n_X_n_. SE, standard error; CI, confidence interval.

## Appendix A. Equations of the Psychoacoustics Metrics

Specific loudness (*N’*)

$$N^{\prime }=0.08{\left(\frac{E_{TQ}}{E_{0}}\right)}^{0.23}\left[{\left(0.5+0.5\frac{E}{E_{TQ}}\right)}^{0.23}-1\right]\left(\frac{\mathrm{sone}}{\mathrm{Bark}}\right)$$

*E*: Excitation of the sounds

*E_TQ_*: Excitation of threshold in quiet

*E*_0_: The reference excitation

Total loudness (*N*)

$$N=\int_{0}^{24\;Bark}N^{\prime }dz\;\left(\mathrm{sone}\right)$$

Sharpness

$$\mathrm{sharpness}=0.11\frac{\int_{0}^{24\;Bark}N^{\prime }g\left(z\right)zdz\;}{\int_{0}^{24\;Bark}N^{\prime }dz}\;\left(\mathrm{acum}\right)$$

$$g\left(z\right)\;=\{\begin{array}{r} 1,\;z\leq 14\\ 0.00012z^{4}-0.0056z^{3}+0.1z^{2}-0.81z+3.51,\;z>14 \end{array}$$

g(z): Critical-band-rate dependent
